# Evaluation optimum ratio of synthetic bone graft material and platelet rich fibrin mixture in a metal 3D printed implant to enhance bone regeneration

**DOI:** 10.1186/s13018-024-04784-y

**Published:** 2024-05-16

**Authors:** Kin Weng Wong, Yu-San Chen, Chun-Li Lin

**Affiliations:** 1 Department of Orthopaedic Surgery, Chi-Mei Medical Center, Tainan, 710 Taiwan; 2 Department of Biomedical Engineering, National Yang Ming Chiao Tung University, 2 No.155, Sec.2, Linong Street, Taipei, 112 Taiwan

**Keywords:** Synthetic bone graft, Platelet rich fibrin, Bone defect, 3D printing, Bone regeneration

## Abstract

**Background:**

This study aims to evaluate the optimal ratio of synthetic bone graft (SBG) material and platelet rich fibrin (PRF) mixed in a metal 3D-printed implant to enhance bone regeneration.

**Methods:**

Specialized titanium hollow implants (5 mm in diameter and 6 mm in height for rabbit; 6 mm in diameter and 5 mm in height for pig) were designed and manufactured using 3D printing technology. The implants were divided into three groups and filled with different bone graft combinations, namely (1) SBG alone; (2) PRF to SBG in 1:1 ratio; (3) PRF to SBG in 2:1 ratio. These three groups were replicated tightly into each bone defect in distal femurs of rabbits (nine implants, n = 3) and femoral shafts of pigs (fifteen implants, n = 5). Animal tissue sections were obtained after euthanasia at the 8th postoperative week. The rabbit specimens were stained with analine blue, while the pig specimens were stained with Masson–Goldner’s trichrome stain to perform histologically examination. All titanium hollow implants were well anchored, except in fracture specimens (three in the rabbit and one fracture in the pig).

**Result:**

Rabbit specimens under analine blue staining showed that collagen tissue increased by about 20% and 40% in the 1:1 ratio group and the 2:1 ratio group, respectively. Masson–Goldner's trichrome stain results showed that new bone growth increased by 32% in the 1:1 ratio PRF to SBG, while − 8% in the 2:1 ratio group.

**Conclusion:**

This study demonstrated that placing a 1:1 ratio combination of PRF and SBG in a stabilized titanium 3D printed implant resulted in an optimal increase in bone growth.

## Background

Recently, advancements in technology have enabled the successful manufacturing of patient-specific (custom) metal implants with intricate, custom shapes for clinical surgery by integrating computed tomography (CT) image reconstruction, computer-aided design (CAD), and metal 3D additive manufacturing (commonly referred to as 3D printing). However, the production of large custom implants often leads to challenges such as bone ingrowth at the metal interface, inadequate implant strength, excessive weight, and induces stress shielding [1–6].

To address these challenges, large custom or geometrically complex metal 3D-printed implants can undergo structural lightweight design during the design stage using topological optimization methods. Structural optimization accompanied hollow spaces within the implant and synthetic or autologous bone grafts can be considered to fill in these spaces to enhances the defect reconstruction and promotes stronger bonding with surrounding bone, ensuring long-term stability after surgery. However, no study has confirmed bone growth efficacy and feasibility between synthetic bone grafts (SBGs) and metal 3D-printed implant.

SBGs are commonly used in orthopedic surgeries to fill up bone defects [7–11]. It provides an osteoconductive effect and is relatively inexpensive. However, the powder or cubic form of the commercialized synthetic bone grafts, which has a large porous architecture, cannot fill up the bone defect/internal space of implant tightly. The loose filling space between the bone graft and the defect/internal implant surfaces could lead to delayed union or non-union [7–11]. Due to the chemical nature of metallic salts, SBGs have been reported to dissolve faster than the bone defect recovery [7–14]. Serious biomechanical problems, such as bone cracks or fracture can easily arise under unfavorable stress in the bone defect area due to the non-integrity structure.

Platelet rich fibrin (PRF) is the second-generation platelet rich concentration. It contains a high amount of growth factors, including platelet-derived growth factor (PDGF), vascular endothelial growth factor (VEGF) and transformation growth factor beta (TGF-β). PRF has already been proven to improve wound healing and enhance tissue regeneration [15, 16]. The jellylike PRF can be mixed with bone graft material to become concentrated growth factor enriched bone graft matrix, also known as sticky bone [17, 18]. This adds the viscosity characteristic to the bone graft, which eases the manipulation of bone grafts during surgery and fills bone defects completely. The polymerized PRF matrix prevents early synthetic bone graft resorption [19]. Therefore, sticky bone is ideal for securely filling irregular traumatic bone defect cavities and internal space of the 3D-printed implant. The sticky bone system has already been applied with dental implants, and has proven to improve bone growth and clinical outcome [20–22]. However, it is still unknown how the mixing ratio of SBG material and PRF can promote good bone regeneration.

When putty-like sticky bone was considered to fill into hollow space within a large metal 3D-printed implant, the implant surface needed to design with window patterns that can allow convenient placement of the filling materials inside the device during surgery. The aim of this study is to evaluate bone graft packing effect, bone ingrowth feasibility and interaction for the PRF composite mixed with different ratio of SBGs (different sticky bone) filled into a relatively small scale hallow titanium 3D-printed implant using in vivo implantation experiments on rabbits and pigs.

## Methods

### 3D-printed implant design and manufacture

**Fig. 1:**
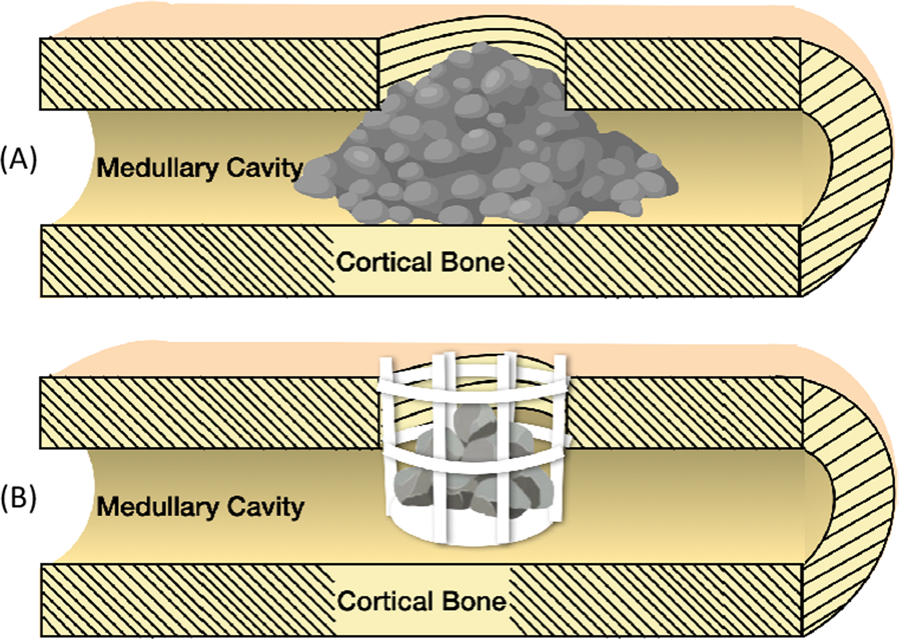
Illustration of 3D-printed implant to prevent bone graft from falling into medullary cavity. **A** without implant **B** with implant

**Fig. 2:**
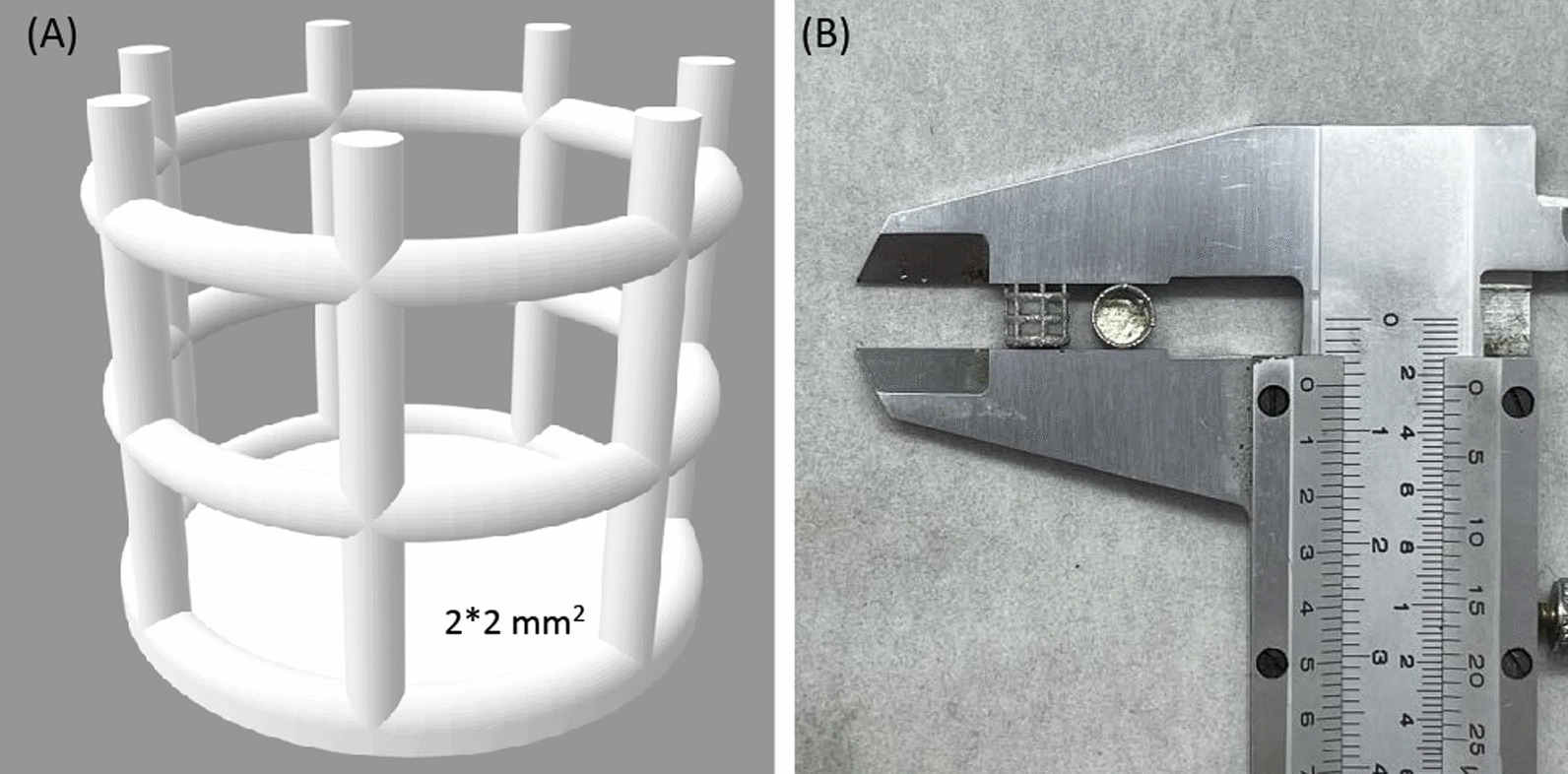
**A** CAD model of 3D-printed implant; **B** metal 3D-printed implant measured by a caliper

Under the intention of preventing sticky bone loss inside the marrow cavity and the fact that bone healing takes place mainly around the cortical bone level, a confining cylinder type implant was designed through computer-aided-design software (PTC Creo, V6.0, PTC Inc., Needham, MA, USA) (Fig. 1). This implant can hold the sticky bone together and be inserted laterally into a rabbit femur condyle or pig femur. The bottom of the cylinder was solid to provide a supporting base. The outer wall surface was a 2 mm square lattice to expose the sticky bone to the patient’s tissue (Fig. 2A). The diameter and height of the cylinder for the rabbit specification was 5 mm and 6 mm, respectively, while it was changed to 6 mm and 5 mm for the pig specification. The implant was then fabricated using a metal 3D printer (AM400, Renishaw, Gloucestershire, UK) with titanium alloy powder (Ti6Al4V powder with average grain size of 30 μm) (Fig. 2B). The titanium powder was selectively scanned and melted by the laser, to form the implant. All Implants were removed from the substrate plate and etched using acid to eliminate residual sandblast particles. Implants were then rinsed with distilled water and cleaned using ultrasonic oscillations before autoclaving.

### PRF and sticky bone production

**Fig. 3:**
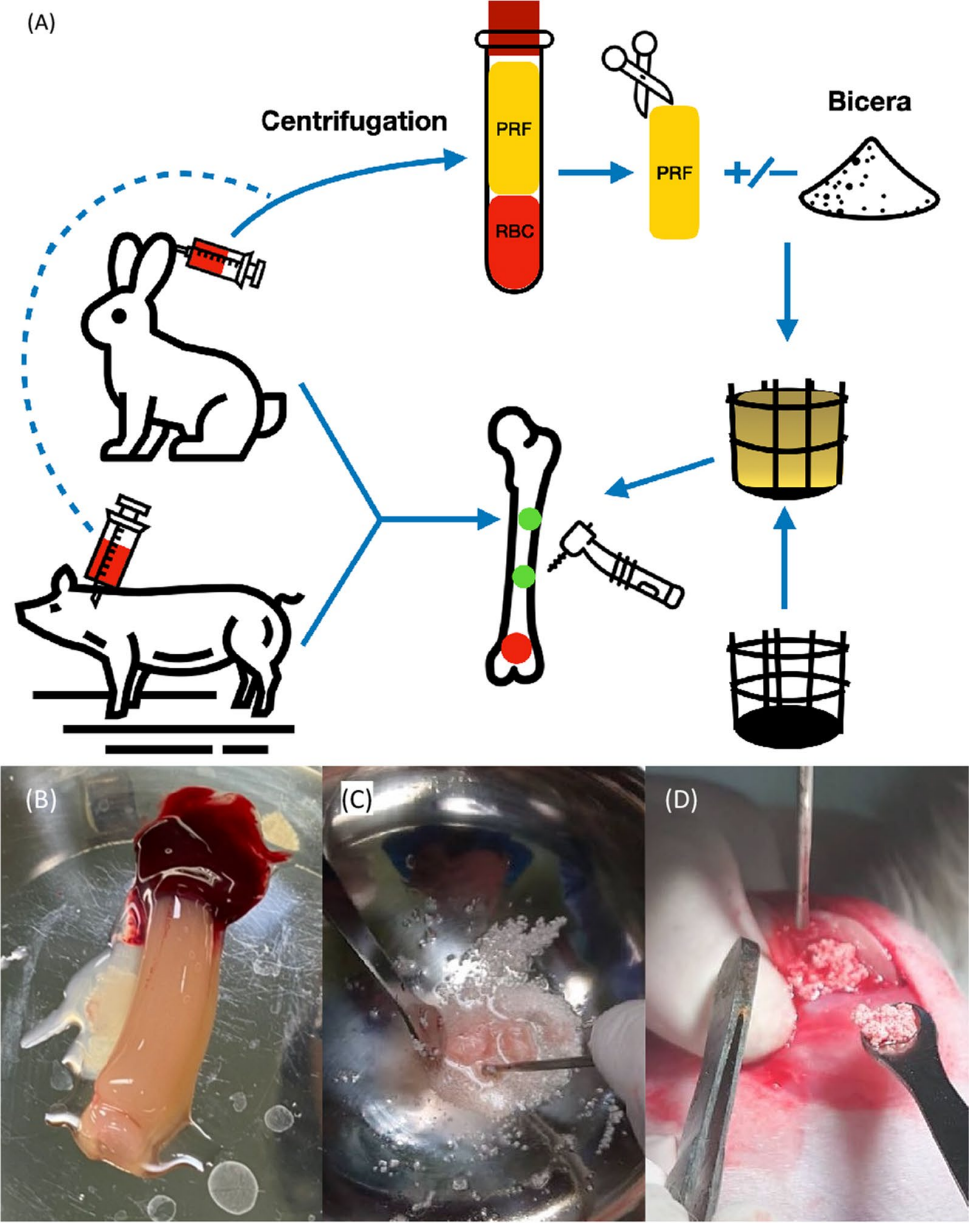
Concept of both rabbits and pig in vivo tests. **A** flow chart of operation procedure, red and green dots on the femur indicate the drill sites on rabbits and pigs, respectively; **B** PRF result from animal blood; **C** sticky bone mixing step; **D** sticky bone operation in the in vivo test

Rabbit and pig target animal venous blood were drawn into a 10 ml plastic serum tube (BD, Vacutainer Plus, Franklin lakes, New Jersey, US) (Fig. 3A). The blood samples were then centrifuged immediately with a compact table top centrifuge (KUBOTA, S300T, Osaka, Japan) under the condition of 2700 rpm for 10 min. The resulted PRF clot was taken out using a surgical tweezer (Fig. 3B). The bottom RBC (red blood cells) clot was cut and discarded. The fibrin clot was then cut into fine pieces and mixed with the designated volume of bone graft material. Continuous mixture was performed until sticky bone formation was completed and filled in the implant (Fig. 3C, D).

### In vivo 3D-printed implant with sticky bone implantation for rabbits

All in vivo experimental procedures were performed using the protocols approved by the Institutional Animal Care and Use Committee of Master Laboratory Co., Ltd. (No.: 21T10-01). Besides, all procedures were conducted in compliance with the ARRIVE (Animal research: reporting of in vivo experiments) guidelines, and all efforts were made to minimize the number of animals and induced pain. Nine female skeletally mature New Zealand rabbits weighing 3.54–3.98 kg (mean ± SD = 3.79 ± 0.17 kg) with average 24 weeks old were used to perform this animal study. Bone mineral density was not calculated because all subjects were young and at a similar age. The rabbit model was chosen due to easier accessibility of bone implantation sites, surgical approach and post-processing analysis that can be used as a basis for larger animals.

**Fig. 4:**
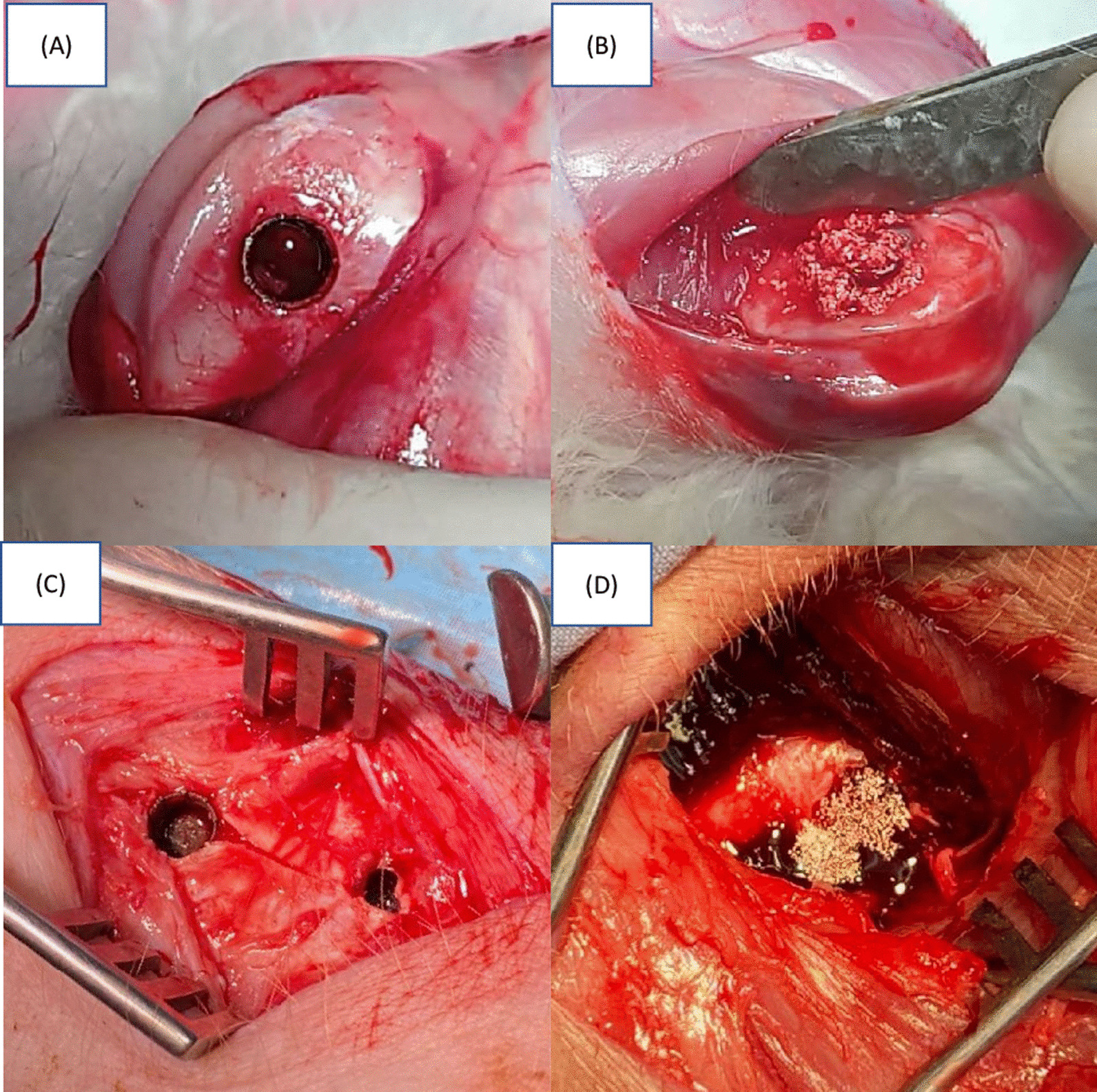
In vivo test implantation results: **A** the metal implant in rabbits; **B** the sticky bone filled in rabbits; **C** the metal implant in pigs; **D** the sticky bone filled in pigs

The rabbit was fasted for 12 h prior to surgery. For sedation and anesthesia, zoletil-50 5 mg/kg (Zoletil, Virbac, Carros, France), xylazine 2 mg/kg (Rompun, Bayer, Leverkusen, North Rhine-Westphalia, Germany), atropine 0.03 mg/kg (Atropine, Nang Kuang Pharmaceutical Co., Ltd., Tainan, Taiwan) and ketoprofen 2 mg/kg (Ketoprofen, Nang Kuang Pharmaceutical Co., Ltd., Tainan, Taiwan) were given by intramuscular injection (IM). Intraoperative analgesia was kept with meloxicam 1 mg/kg (Achefree, Swiss Pharmaceutical Co. Ltd, Tainan, Taiwan), subcutaneous injection if needed. After sedation, venous blood was drawn from a marginal ear vein, and sticky bone was produced according to the previous description (Fig. 4A, B).

The rabbit was then put in the supine position, with a 2–3 cm skin incision made at the right hind knee area to reveal the femur bone lateral condyle. A 5 mm-diameter hole was predrilled and a 3D printing implant 5 mm diameter and 6 mm height was tightly implanted into the hole. The implant was then filled with three designated PRF mixed with SBGs (Bicera bone graft substitute–pore size: 300–600 μm, Wiltrom, Hsinchu, Taiwan), i.e. (1) only SBG; (2) PRF with SBG in 1:1 ratio; (3) PRF with SBG in 2:1 ratio. Each combination was replicated in 3 different rabbits (n = 3) (Fig. 4A, B). Finally, the wound was closed in layers without a visible bleeding point. Topical antibiotic, penicillin 3000iu/kg (Penicillin G procaine, Ta Fong, Pharmaceutical Co., Ltd., Changhua, Taiwan), was applied prior to wound closure to control surgical site infection. Prophylactic antibiotics with enrofloxacin 5 mg/kg (BAYTRIL, Bayer, Leverkusen, North Rhine-Westphalia, Germany), was administered IM, QD for 5 days. Once analgesia was no longer required, the animals were monitored once daily. Surgical site X-ray images were taken at 4th and 8th week after surgery. After 8 weeks, under deep general anesthesia, the rabbits were euthanized with heart exsanguination. The right femur was removed and preserved in 4% formaldehyde solution.

### In vivo 3D-printed implant with sticky bone implantation for pigs

The in vivo pig study was also reviewed and approved by the same committee of the IACUC (No.: 110040601). Four female skeletally mature Yorkshire pigs, weighing around 30 kg (Mean ± SD: 32.5 ± 2.78 kg) and aged 3 months old on average, were used to perform this animal study. This pig model was chosen due to its similarity in bone quality, density, anatomy and size compared with the human femur. Pig femurs were also lower in variability and cost. Bone mineral density was also not calculated in this study because all pigs were young and at the same age (3 months ± 1 week).

The pigs were also fasted for 12 h prior to surgery. The zoletil-50 5 mg/kg, xylazine 1 mg/kg, and atropine 0.03 mg/kg were given by IM for sedation and anesthesia. Anesthesia was maintained with 3% isoflurane (Attane, Panion & BF biotech, Taipei Taiwan) in endotracheal inhalation with oxygen 2 L/min. After sedation, venous blood was drawn from the subclavian vein, and sticky bone was produced as mentioned above. The pig was then placed in the lateral position. The skin was disinfected with Povidone-iodine. Two 3–4 cm skin incisions were made at the bilateral hind limbs, respectively. Superficial muscles were separated properly with bleeding control. The femur bone shaft was then revealed (Fig. 4C, D).

Two implants were implanted into two corresponding 6 mm predrilled holes on each hind limb without overlapping and falling into the marrow cavity. The same three combinations of PRF mixed with synthetic bone graft, i.e. only SBG, PRF with SBG in 1:1 ratio and PRF with SBG in 2:1 ratio was then filled into the implants. Each combination was replicated at least 5 times (n = 5) and scattered randomly among all drilled holes (there were totally 16 holes) to eliminate the within‑combination variance (Fig. 4C, D). The same wound care and X-ray images were taken at the same time point as the rabbits after surgery. After 8 weeks the pigs were sacrificed and bilateral femur bones were removed and preserved in 4% formaldehyde solution for further processing.

### Histomorphometrical evaluation

**Fig. 5:**
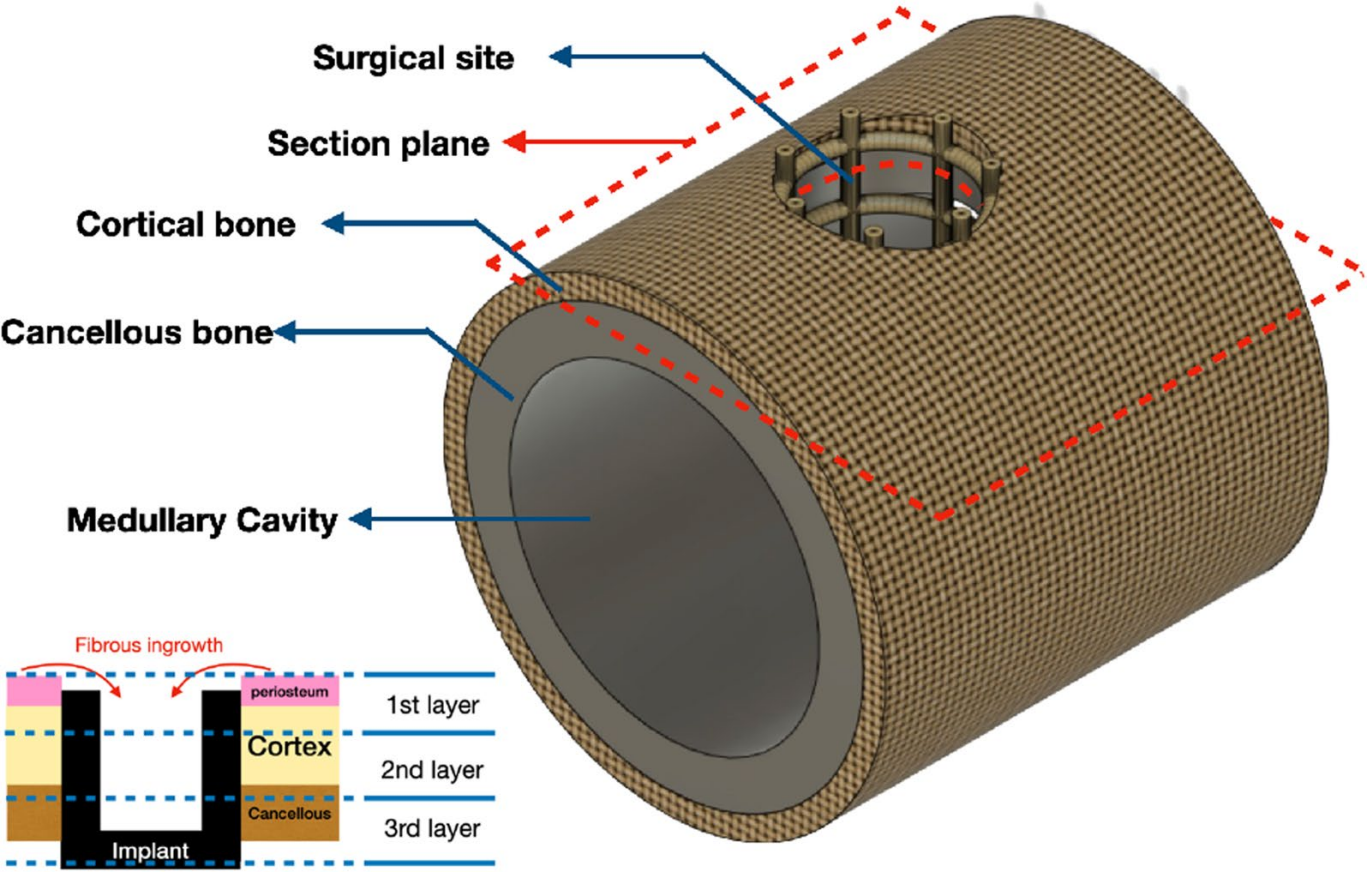
Indication of sectioning in hard tissue histology. Section level was determined right below the cortical bone level

Each implant and its surrounding hard tissue were sectioned and dehydrated in a graded series of alcohol (20–40–60–80–100%). The sample was then embedded and sliced paralleled to the cortical bone level (Fig. 5). Each section was taken every 1.5 mm (included cutter thickness) to ensure complete histological presentation and each specimen can be cut up to three slices (Fig. 5). The section above and below marked level were covered with fibrous tissue and metallic bottom of implant, respectively. Therefore, these two sections have no presentational significance. After attachment to glass slides the samples were finely ground to the desired thickness for histochemical staining.

The rabbit ground bone sample was only stained in blue using analine blue solution to identify tissue healing inside the basket implant. The pig bone sample was stained using Masson–Goldner’s trichrome stain (Masson–Goldner staining kit, Merck & Co, Kenilworth, New Jersey, US) to identify different types of healing tissue inside the basket implant.

After staining, all slides were rinsed with 70%, 90%, then 100% alcohol, and properly sealed for photographing. Images were taken under 12.5× magnifications and analyzed using image processing software (ImageJ 1.53a for MacOS, National Institutes of Health, USA). A cylindrical cut area was selected in each sample. The unnecessary areas were cut away and removed. The black area found under light microscope was represented the metal material of the 3D-printed hollow implant. The blue area for the rabbit and red/yellow/green area for pig were represented soft/hard tissue cell ingrowth. Therefore, target colors’ (blue/red/yellow/green) areas were selected using hue adjustment in the color threshold function and area ratios of various target colors to black deductions were calculated. The blue area ratio for rabbit and green area ratio for pig of the SGB group were then treated as the control group to calculate the relative percentages of different combinations of PRF for bone ingrowth efficiency.

## Results

The body weights of both animal models were recorded, and no drastic variation was found before sacrificing. No surgical wound infection or other complications were found at the 8th week. All animals were euthanized as scheduled. The represented x rays of intact and fractured rabbit and pig bones are shown in Tables 1 and 2, respectively. As the X-ray images show, three fractures were found among the nine rabbit bones and one fracture was found in each of the three different PRF combination groups (SBG, PRF:SBG = 1:1 and PRF:SBG = 2:1). Only one fracture was found in the pure synthetic bone graft group in the pig test.

**Table 1:**
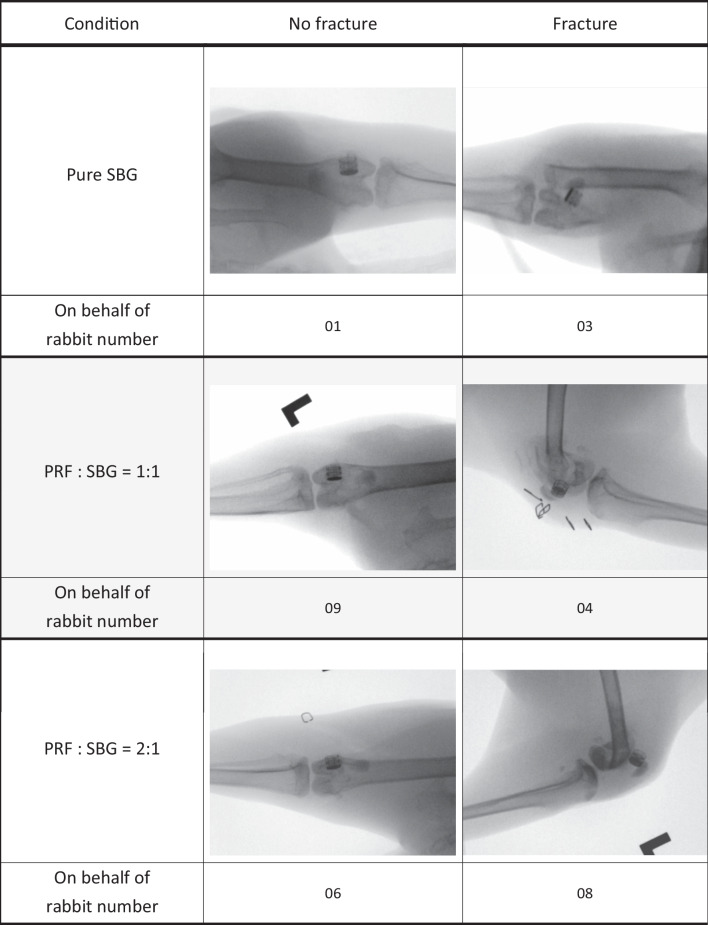
Record of in vivo rabbit test, experimental conditions and representative X rays

**Table 2:**
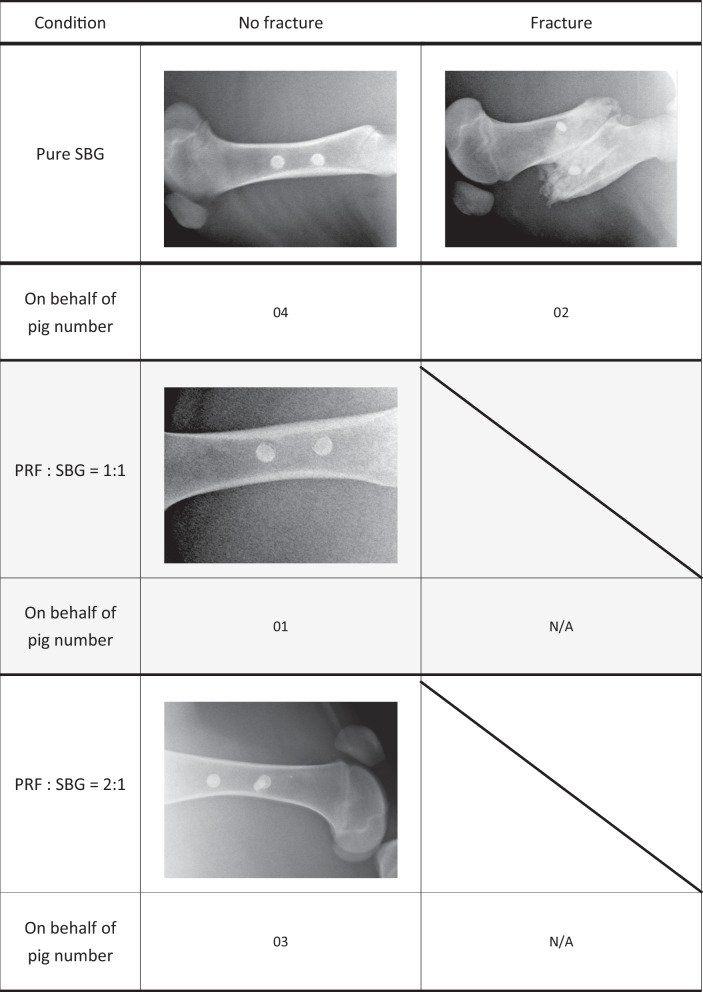
Record of in vivo pig test, experimental conditions and representative X rays

**Fig. 6:**
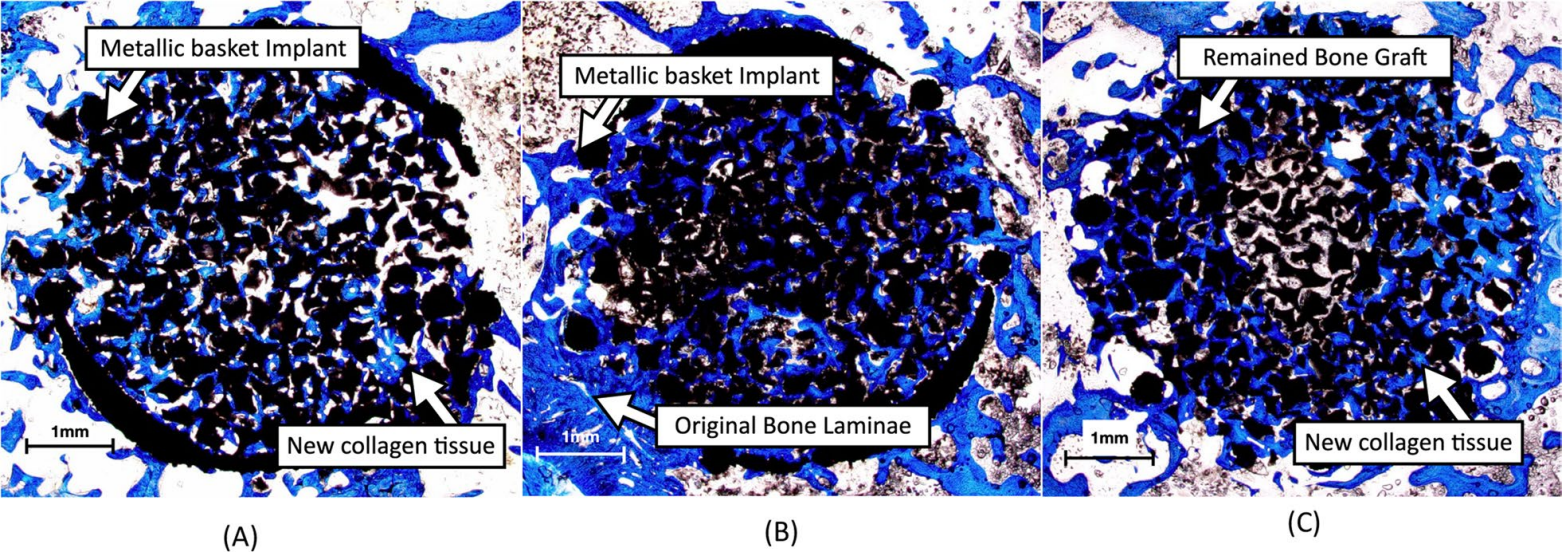
Histology of the rabbit in vivo test, staining with analine blue. Blue color indicating collagen (including bone matrix, smooth muscle and fibrous tissue). Conditions: **A** SBG only; **B** PRF with SBG in 1:1; **C** PRF with SBG in 2:1

In the rabbit histomorphometrical evaluation, blue color indicating the amount of collagen-tissue and bone growth (Fig. 6). Percentage ratios of blue color to black deduction areas were 52.73%, 63.09% and 73.80% for SBG, PRF:SBG = 1:1 and PRF:SBG = 2:1 groups, respectively (Table 3). This result indicated that SBG mixed with higher PRF ratios presented relative higher percentage of collagen-tissue/bone growth. The increased percentage were found 19.65% (about 20%) and 39.95% (about 40%) for PRF with SBG in 1:1 and 2:1 ratio, respectively (Table 3).

**Table 3:** Histology analysis of in vivo test of rabbit. Data are mean value

Condition	Blue area	Calculated efficiency
Calculation	Blue color area/(Total area − Black area) (%)	Target combination/SBG % (%)
SBG	52.73	100
PRF with SBG in 1:1	63.09	119.65
PRF with SBG in 2:1	73.80	139.95

**Fig. 7:**
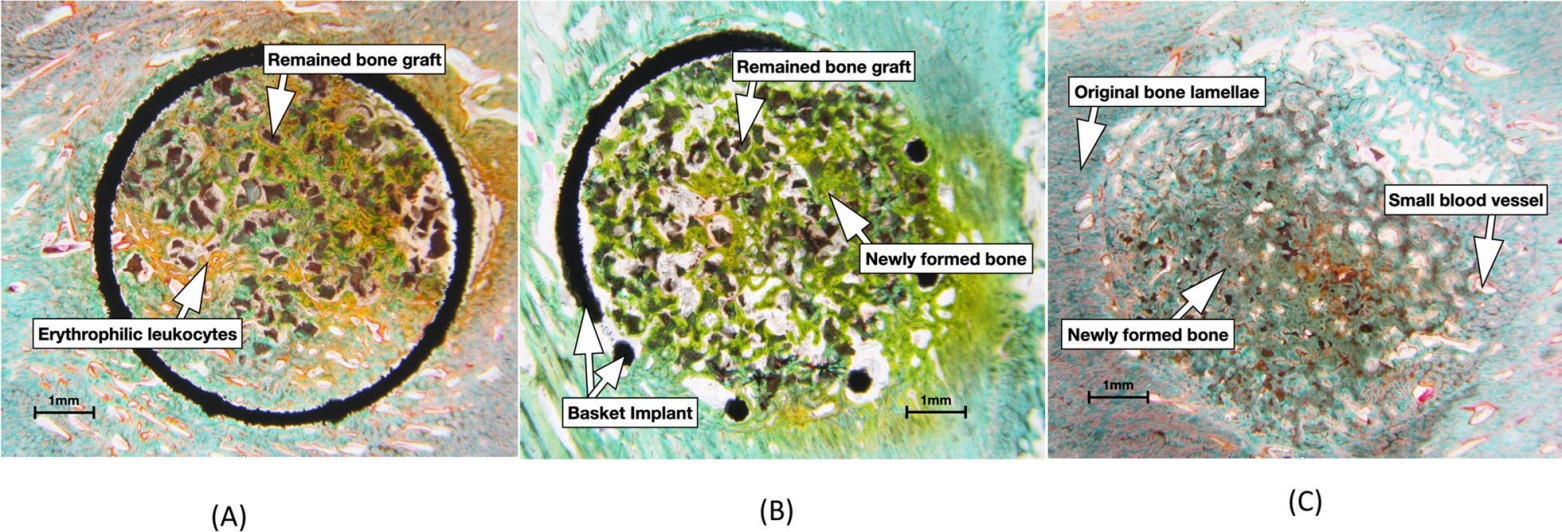
Histology of the pig in vivo test, staining with Masson–Goldner's trichrome stain. Connective tissue (bone tissue) stained green, muscle tissue stained red, erythrocyte stained yellow. Conditions: **A** SBG only; **B** PRF with SBG in 1:1; **C** PRF with SBG in 2:1

We applied the Masson–Goldner’s trichrome stain for the pig test and found by which the cancellous bone was stained in green, the muscle-related tissue (outer-periosteum) was stained in red, and the erythrophilic leukocytes were stained in yellow (Fig. 7). Therefore, the expected results showed bone growth trend in pigs was different from that in rabbits. The increased percentages of bone growth were 32.13% for PRF with SBG in 1:1 combination and − 8.4% for the 2:1 group when compared to the pure SBG (Table 4).

**Table 4:** Histology analysis of in vivo pig test

Calculation	Target color area/(Total area − Black area)		Target combination (Green color) /SBG %	
Condition	Red area	Yellow area	Green area	Calculated efficiency (% of bone growth) (%)
SBG	39.30% (± 19.2%)	32.00% (± 15.4%)	25.41% (± 22.1%)	100
PRF with SBG in 1:1	22.57% (± 13.2%)	42.87% (± 6.1%)	33.58% (± 10.3%)	132.13
PRF with SBG in 2:1	37.32% (± 12.7%)	37.99% (± 17.3%)	23.28% (± 8.1%)	91.60

## Discussion

The SBG is a variety of bone substitutes are used in orthopedic operations. In this study, we adapted Bicera as a representative SBG because it is one of the most commonly used bone substitute material [23]. Furthermore, it is available in powder and can be access conveniently. The chemical components of Bicera are hydroxyapatite (60%) and ß-tricalcium phosphate (40%), both compounds were widely used as SBGs [24, 25]. The porous structure of Bicera can provide better adhering sites for PRF in forming sticky bone [26]. These features can both be found in many commercialized synthetic bone grafts. Therefore, the role of Bicera in this study can be replaced by other bone graft materials that possess similar chemical components and physical characteristics. The role of SBG material can be further replaced by autologous bone grafts, which is more ideal in promoting bone healing [27]. In the dental implanting procedure, autologous bone grafts can be obtained during the bone drilling process. Dentistry cases report on sticky bone mixed with autologous bone graft material with PRF [28]. This combination may be adapted to our implanting system in future studies.

Specific bioactive additives, such as PRP, PRGF, PRF techniques have been found to promote the capability to accelerate wound healing, regulate inflammation, and improve soft and hard tissue regeneration [29–36]. The advantages of these bioactive additives are its autologous nature, simple collection, ease of chair-side preparation, and simple clinical application without the risks associated with allogeneic or animal derived products. Compared to the PRP, the PRF exists advantages with greater simplicity of production, absence of blood manipulation, no additives, contains more healing factors and more stem cells, less trauma. The biological properties of PRF clearly show an interesting surgical versatility and all the characteristics that can support faster tissue regeneration and high-quality clinical outcomes. The PRF is able to stimulate osteogenesis in bone environment, in addition to angiogenesis [31–34]. The pictures in Fig. 3B–D demonstrate successful PRF and sticky bone production for the rabbits and pigs. Although reports exist on achieving PRF and sticky bone production for other types of animals [37, 38], the ideal PRF and synthetic bone graft ratio has not yet been tested.

Theoretically, higher growth factor concentration should result in faster tissue growth, according to growth factor physiology. Therefore, we tested the sticky bone combination (PRF:SBG) in 2:1 and 1:1 ratio in the rabbit in vivo test. This test determined whether the PRF, synthetic SBG and metallic holow implant combination could provide a positive effect in bone healing. The result in Table 3 indicates the positive effect from the addition of PRF and a proportional relation between the collagen-tissue and growth. When compared to the SBG group alone, the collagen growth (including bone and soft tissue) was enhanced about 20% at 1:1 ratio while as high as 40% enhancement was noted from the 2:1 ratio. However, different types of collagen would be secreted by fibroblasts, osteoblasts, and other inflammatory cells in the bone healing process. With analine blue used in rabbit tests to bind collagen in the bone matrix, smooth muscle and fibrous tissue, this result is prone to misjudgment. The blue dye area within the basket implant could be easily misunderstood as the amount of new bone growth.

Based on the rabbit in vivo test, we designed a subsequent test using Yorkshire pigs with larger bone size to expand the sample size (n = 5). Moreover, we adapted another histochemical stain the Masson–Goldner’s trichrome staining to identify differentiated tissue growing inside the hollow implant [39]. Under trichrome staining, small blood vessels were marked with red color, and found around the implant site under light microscopy, as shown in Fig. 7. The neovascularization is part of wound healing and can be enhanced by VEGF, PDGF and other factors inside PRF. Accordingly the percentage of red area is proportional to the PRF ratio, coupled with a decrease in the percentage of green area. Otherwise, leukocytes (white blood cells) stained with yellow color were found surrounding the new-grown bone tissue and the residual synthetic bone graft. This co-staining phenomenon indicated synthetic bone graft degradation, which was reported to attract erythrophilic leukocytes [25]. Newly formed lamellar bone by which was stained in green within the implants.

This finding indicated that PRF growth factors could favor in enhancing fibro epithelial tissue growth rather than bone tissue in higher concentrations. Therefore, we are able to explain the discordant results between the rabbit and pig tests. Since analine blue could bind to collagen in the bone matrix, smooth muscle and fibrous tissue, the higher percentage of blue area in the 2:1 group might represent higher vessel growth in rabbit testing. After differentiating muscle tissue from bone tissue using trichrome staining, we can conclude that sticky bone with 1:1 portion of PRF and synthetic bone graft provided better bone healing effect in the pig test.

The titanium 3D printing basket implants with outer surface lattices were used as a confining boundary to support the sticky bone structure stability before bone union. Despite femur bone fractures in three rabbits and one pig, the titanium baskets in all test subjects, including those with fractures, remained intact under X ray at the 4th and 8th weeks. This proves that the titanium basket implant was stable and strong enough to withstand the test. However, larger defects reconstructed with specific 3D printing implants may require plate-and-screw fixation to enhance the structural strength to ensure bone union in future studies [3, 40]. Bicera was chosen as the representative SBG in this study. Other types of bone grafts such as autograft, allograft or other commercial products with different components might have different results. In our study, due to the small size of the implanted SBG, it was not easy to measure the accurate absorption rate. However, it can be estimated from histological sections. Since the Bicera we used is opaque, we could measure the black area to estimate the remaining SBG. In PRF with SBG 1:1 group of the pig experiment, each sample resulted in around 4.6 mm^2^ of black area (excluded metal bracket area), however, area occupied by SBG accounts for only half of the total area (28.26 mm^2^). Therefore, the proportion of SBG that still exists should be about 33% (4.6 mm^2^/14.13 mm^2^). Through such calculation we can probably estimate the resorption rate was around 67%. Our study is a single cross section histomorphometrical evaluation. Serial histomorphometrical healing process evaluations were not included.

## Conclusion

This study successfully applied Masson’s Goldner trichrome stain to indicate that equal parts of PRF and SBG mixtures (1:1) in the titanium 3D printed hollow implant can obtain the best increased bone growth of 32.13% in the pig graft experiment.

## Data Availability

The datasets analyzed during the current study are available from the corresponding author on reasonable request.

## Abbreviations

SBG: Synthetic bone graft
PRF: Platelet rich fibrin
CT: Computed tomography
CAD: Computer-aided design
PDGF: Platelet-derived growth factor
VEGF: Vascular endothelial growth factor
TGF-β: Transformation growth factor beta

## References

[1] Wong KC (2016). 3D-printed patient-specific applications in orthopedics. Orthop Res Rev.

[2] Li CH, Wu CH, Lin CL (2020). Design of a patient-specific mandible reconstruction implant with dental prosthesis for metal 3D printing using integrated weighted topology optimization and finite element analysis. J Mech Behav Biomed Mater.

[3] Wu PK, Lee CW, Sun WH, Lin CL (2021). Biomechanical analysis and design method for patient-specific reconstructive implants for large bone defects of the distal lateral femur. Biosensors (Basel).

[4] Lin CL, Wang YT, Chang CM, Wu CH, Tsai WH (2021). Design criteria for patient-specific mandibular continuity defect reconstructed implant with lightweight structure using weighted topology optimization and validated with biomechanical fatigue testing. Int J Bioprint.

[5] Li Z, Lu M, Min L, Luo Y, Tu C (2023). Treatment of pelvic giant cell tumor by wide resection with patient-specific bone-cutting guide and reconstruction with 3D-printed personalized implant. J Orthop Surg Res.

[6] Migliorini F, Padula GL, Torsiello E, Spiezia F, Oliva F, Maffulli N (2021). Strategies for large bone defect reconstruction after trauma, infections or tumour excision: a comprehensive review of the literature. Eur J Med Res.

[7] Shahrezaie M, Moshiri A, Shekarchi B, Oryan A, Maffulli N, Parvizi J (2018). Effectiveness of tissue engineered three-dimensional bioactive graft on bone healing and regeneration: an in vivo study with significant clinical value. J Tissue Eng Regen Med.

[8] Oryan A, Alidadi S, Moshiri A, Maffulli N (2014). Bone regenerative medicine: classic options, novel strategies, and future directions. J Orthop Surg Res.

[9] Oliva F, Migliorini F, Cuozzo F, Torsiello E, Hildebrand F, Maffulli N (2021). Outcomes and complications of the reamer irrigator aspirator versus traditional iliac crest bone graft harvesting: a systematic review and meta-analysis. J Orthop Traumatol.

[10] Migliorini F, Cuozzo F, Torsiello E, Spiezia F, Oliva F, Maffulli N (2021). Autologous bone grafting in trauma and orthopaedic surgery: an evidence-based narrative review. J Clin Med.

[11] Fernandez de Grado G, Keller L, Idoux-Gillet Y, Wagner Q, Musse A-M, Benkirane-Jessel M, Offner D (2018). Bone substitutes: a review of their characteristics, clinical use, and perspectives for large bone defects management. J Tissue Eng.

[12] Buser Z, Brodke DS, Youssef JA, Meisel HJ, Myhre SL, Hashimoto R, Park JB, Yoon ST, Wang JC (2016). Synthetic bone graft versus autograft or allograft for spinal fusion: a systematic review. J Neurosurg Spine.

[13] Hanke A, Baumlein M, Lang S, Gueorguiev B, Nerlich M, Perren T, Rillmann P, Ryf C, Miclau T, Loibl M (2017). Long-term radiographic appearance of calcium-phosphate synthetic bone grafts after surgical treatment of tibial plateau fractures. Injury.

[14] Kobayashi H, Kageyama Y, Shido Y (2017). Calcaneocuboid distraction arthrodesis with synthetic bone grafts: preliminary results of an innovative bone grafting procedure in 13 patients. J Foot Ankle Surg.

[15] Nishimoto S, Fujita K, Sotsuka Y, Kinoshita M, Fujiwara T, Kawai K, Kakibuchi M (2015). Growth factor measurement and histological analysis in platelet rich fibrin: a pilot study. J Maxillofac Oral Surg.

[16] Miron RJ, Zucchelli G, Pikos MA, Salama M, Lee S, Guillemette V, Fujioka-Kobayashi M, Bishara M, Zhang Y, Wang HL, Chandad F, Nacopoulos C, Simonpieri A, Aalam AA, Felice P, Sammartino G, Ghanaati S, Hernandez MA, Choukroun J (2017). Use of platelet-rich fibrin in regenerative dentistry: a systematic review. Clin Oral Investig.

[17] Bolukbasi N, Yeniyol S, Tekkesin MS, Altunatmaz K (2013). The use of platelet-rich fibrin in combination with biphasic calcium phosphate in the treatment of bone defects: a histologic and histomorphometric study. Curr Ther Res Clin Exp.

[18] Bolukbasi N, Ersanli S, Keklikoglu N, Basegmez C, Ozdemir T (2015). Sinus augmentation with platelet-rich fibrin in combination with bovine bone graft versus bovine bone graft in combination with collagen membrane. J Oral Implantol.

[19] Schubert T, Lafont S, Beaurin G, Grisay G, Behets C, Gianello P, Dufrane D (2013). Critical size bone defect reconstruction by an autologous 3D osteogenic-like tissue derived from differentiated adipose MSCs. Biomaterials.

[20] Agrawal AA (2017). Evolution, current status and advances in application of platelet concentrate in periodontics and implantology. World J Clin Cases.

[21] Soni R, Priya A, Yadav H, Mishra N, Kumar L (2019). Bone augmentation with sticky bone and platelet-rich fibrin by ridge-split technique and nasal floor engagement for immediate loading of dental implant after extracting impacted canine. Natl J Maxillofac Surg.

[22] Scarano A, Inchingolo F, Murmura G, Traini T, Piattelli A, Lorusso F (2018). Three-dimensional architecture and mechanical properties of bovine bone mixed with autologous platelet liquid, blood, or physiological water: an in vitro study. Int J Mol Sci.

[23] Calori GM, Mazza E, Colombo M, Ripamonti C (2011). The use of bone-graft substitutes in large bone defects: Any specific needs?. Injury.

[24] Ebrahimi M, Botelho MG, Dorozhkin SV (2016). Biphasic calcium phosphates bioceramics (HA/TCP): concept, physicochemical properties and the impact of standardization of study protocols in biomaterials research. Mater Sci Eng C Mater Biol.

[25] Bohner M, Santoni BLG, Dobelin N (2020). Beta-tricalcium phosphate for bone substitution: synthesis and properties. Acta Biomater.

[26] Lekovic V, Milinkovic I, Aleksic Z, Jankovic S, Stankovic P, Kenney EB, Camargo PM (2012). Platelet-rich fibrin and bovine porous bone mineral vs. platelet-rich fibrin in the treatment of intrabony periodontal defects. J Periodontal Res.

[27] Fontes Martins LC, Sousa Campos de Oliveira AL, Aloise AC, Scavone de Macedo LG, Teixeira ML, Moy PK, Pelegrine AA (2021). Bone marrow aspirate concentrate and platelet-rich fibrin in fresh extraction sockets: a histomorphometric and immunohistochemical study in humans. J Craniomaxillofac Surg.

[28] Andrade C, Camino J, Nally M, Quirynen M, Martinez B, Pinto N (2020). Combining autologous particulate dentin, L-PRF, and fibrinogen to create a matrix for predictable ridge preservation: a pilot clinical study. Clin Oral Investig.

[29] Giannini S, Cielo A, Bonanome L, Rastelli C, Derla C, Corpaci F, Falisi G (2015). Comparison between PRP, PRGF and PRF: lights and shadows in three similar but different protocols. Eur Rev Med Pharmacol Sci.

[30] Castro AB, Meschi N, Temmerman A, Pinto N, Lambrechts P, Teughels W, Quirynen M (2017). Regenerative potential of leucocyte- and platelet-rich fibrin. Part A: intra-bony defects, furcation defects and periodontal plastic surgery. A systematic review and meta-analysis. J Clin Periodontol.

[31] Miron RJ, Dham A, Dham U, Zhang Y, Pikos MA, Sculean A (2019). The effect of age, gender, and time between blood draw and start of centrifugation on the size outcomes of platelet-rich fibrin (PRF) membranes. Clin Oral Investig.

[32] Miron RJ, Xu H, Chai J, Wang J, Zheng S, Feng M, Zhang X, Wei Y, Chen Y, Carlos Fernando de Almeida Barros Mourão C, Sculean A, Zhang Y (2020). Comparison of platelet-rich fibrin (PRF) produced using 3 commercially available centrifuges at both high (~ 700 g) and low (~ 200 g) relative centrifugation forces. Clin Oral Investig.

[33] Dohan Ehrenfest DM, Pinto NR, Pereda A, Jiménez P, Del Corso M, Kang BS, Nally M, Lanata N, Wang HL, Quirynen M (2018). The impact of the centrifuge characteristics and centrifugation protocols on the cells, growth factors, and fibrin architecture of a leukocyte- and platelet-rich fibrin (L-PRF) clot and membrane. Platelets.

[34] Li GY, Yin JM, Ding H, Jia WT, Zhang CQ (2013). Efficacy of leukocyte- and platelet-rich plasma gel (L-PRP gel) in treating osteomyelitis in a rabbit model. J Orthop Res.

[35] Kobayashi E, Fluckiger L, Fujioka-Kobayashi M, Sawada K, Sculean A, Schaller B, Miron RJ (2016). Comparative release of growth factors from PRP, PRF, and advanced-PRF. Clin Oral Investig.

[36] Masuki H, Okudera T, Watanebe T, Suzuki M, Nishiyama K, Okudera H, Nakata K, Su CY, Kawase T (2016). Growth factor and pro-inflammatory cytokine contents in platelet-rich plasma (PRP), plasma rich in growth factors (PRGF), advanced platelet-rich fibrin (A-PRF), and concentrated growth factors (CGF). Int J Implant Dent.

[37] Liu R, Long Y, Liu L, Zhao X (2020). Effect of platelet-rich fibrin on fat grafting in animal models: a meta-analysis. Aesthet Plast Surg.

[38] Lee YK, Wadhwa P, Cai H, Jung SU, Zhao BC, Rim JS, Kim DH, Jang HS, Lee ES (2021). Micro-CT and histomorphometric study of bone regeneration effect with autogenous tooth biomaterial enriched with platelet-rich fibrin in an animal model. Scanning.

[39] Rentsch C, Schneiders W, Manthey S, Rentsch B, Rammelt S (2014). Comprehensive histological evaluation of bone implants. Biomatter.

[40] Wong KW, Wu CD, Chien CS, Lee CW, Yang TH, Lin CL (2020). Patient-specific 3-dimensional printing titanium implant biomechanical evaluation for complex distal femoral open fracture reconstruction with segmental large bone defect: a nonlinear finite element analysis. Appl Sci.

